# Sodium Oxybate as a Potential New Treatment for Catatonia in Patients With Depression, Bipolar Disorder, or a Psychotic Disorder: Protocol for a Randomized Controlled Trial

**DOI:** 10.2196/68356

**Published:** 2025-07-24

**Authors:** Lilian Bot, Josine G van Mill, Laetitia J C A Smarius, Adriaan W Hoogendoorn, Bram W C Storosum, Christiaan H Vinkers, Gabriel E Jacobs, Gert Jan Lammers, Hanna M Heller, Jantine C A Colen-de Koning, Joris J B van der Vlugt, Marloes S Oudijn, Martijn S van Noorden, Pierre M Bet, Rolf Fronczek, Heleen S van der Heijden, Sjors M M Lange, Ysbrand D van der Werf, Eric R van Exel

**Affiliations:** 1 Department of Psychiatry Amsterdam University Medical Centers Amsterdam The Netherlands; 2 Specialized Mental Health Care GGZ InGeest Amsterdam The Netherlands; 3 Amsterdam Public Health (Mental Health Program) Amsterdam University Medical Centers Amsterdam The Netherlands; 4 Specialized Mental Health Care Arkin Amsterdam The Netherlands; 5 Compulsivity Impulsivity and Attention Amsterdam Neuroscience Amsterdam The Netherlands; 6 Department of Psychiatry Leiden University Medical Center Leiden The Netherlands; 7 Department of Psychiatry Research Centre for Human Drug Research Leiden The Netherlands; 8 Department of Neurology Leiden University Medical Center Leiden The Netherlands; 9 Expertise Centre for Epilepsy and Sleep Medicine Stichting Epilepsie Instellingen Nederland Heemstede The Netherlands; 10 Department of Clinical Pharmacology and Pharmacy Amsterdam University Medical Centers Amsterdam The Netherlands; 11 Department of Psychiatry Molenaar Antes - Parnassiagroep Rotterdam The Netherlands; 12 Amsterdam Public Health (Personalized Medicine Program) Amsterdam University Medical Centers Amsterdam The Netherlands; 13 Specialized Mental Health Care GGZ Centraal Hilversum The Netherlands; 14 Department of Anatomy and Neurosciences Amsterdam University Medical Centers Vrije Universiteit Amsterdam Amsterdam The Netherlands

**Keywords:** catatonia, sodium oxybate, GABA, lorazepam, gamma-aminobutyric acid

## Abstract

**Background:**

Catatonia is a severe psychomotor syndrome predominantly associated with depressive, bipolar, and psychotic disorders. Untreated catatonia has a 10% mortality rate and may lead to complications such as renal failure, rhabdomyolysis, pneumonia, embolism, and contractures. High doses of the benzodiazepine lorazepam, a gamma-aminobutyric acid (GABA)-A receptor modulator, are the primary pharmacological treatment, enhancing GABA’s inhibitory effect, potentially reducing symptoms of catatonia. However, lorazepam is ineffective in about 25% of cases, leaving electroconvulsive therapy (ECT) as the only well-investigated alternative. Although often effective, ECT may have severe side effects and is not easily accepted among patients and caregivers. Therefore, there is an urgent need for novel therapies for catatonia. Sodium oxybate, a GABA precursor and GABA-B receptor agonist, is a promising alternative treatment based on observational data, but its efficacy has never been thoroughly investigated.

**Objective:**

This study aims to evaluate the efficacy and safety of sodium oxybate in treating catatonia unresponsive to lorazepam, while also capturing the natural course and determinants of catatonia through an observational cohort.

**Methods:**

The Laborit trial consists of a cohort study and an embedded single-blind randomized controlled trial (RCT). Patients with catatonia admitted to a psychiatric ward may join the study’s cohort, where their clinical characteristics are recorded. Standard care, including lorazepam up to 24 mg/day, will be administered. On day 4, the Bush Francis Catatonia Rating Scale (BFCRS) will be used to measure symptom response. Patients with ≤50% improvement on the BFCRS, compared to the score at start, will be eligible for the trial. A total of 42 patients will be randomly assigned to either the sodium oxybate group, after a 2-day lorazepam reduction period, or the continuation of lorazepam. The primary endpoint is response, measured by the BFCRS score change after 4 days of treatment. A ≥50% reduction in BFCRS will define a responder, who will continue allocated treatment for an additional 10 days, with a secondary endpoint at 14 days. Data will be analyzed using intention-to-treat and per-protocol methods, with chi-square and logistic regression tests to compare group response and remission rates.

**Results:**

This study was funded by the Dutch Brain Foundation (Hersenstichting) in December 2020. The study protocol was approved by the Amsterdam UMC Ethics Board on May 22, 2023. As of March 2025, the first 4 participants have been included in the cohort, with no trial participants enrolled yet.

**Conclusions:**

If positive, the results of this RCT may pave the way for international catatonia researchers and clinicians to introduce a new pharmacological treatment option for catatonia. Implementation could potentially benefit patients who endure this severe syndrome and present health care professionals with an additional treatment option.

**Trial Registration:**

ISRCTN ISRCTN11236443; https://tinyurl.com/4px5s4aa

**International Registered Report Identifier (IRRID):**

PRR1-10.2196/68356

## Introduction

### Background

Catatonia was described by Kahlbaum in 1874 as a syndrome accompanied by different psychiatric and somatic symptoms [1,2]. This severe condition has a prevalence of at least 10% in psychiatric hospitals [3,4]. Catatonia is strongly associated with psychiatric disorders, where 30% of cases of catatonia are associated with schizophrenia, and 43% have an underlying bipolar disorder [1]. In addition, catatonia has been linked to other psychiatric disorders such as depressive disorder, obsessive-compulsive disorder, posttraumatic stress disorder, and withdrawal from alcohol or benzodiazepines [1]. There are several somatic underlying disorders to catatonia, such as encephalitis or autoimmune disorders [5]. Even though there are no well-defined risk factors for the development of catatonia, it is known that antipsychotics—especially those with high dopamine receptor affinity—may lead to the development of catatonia [5]. It is hypothesized that catatonia is caused by either a dysfunction of gamma-aminobutyric acid (GABA) receptors or a deficit in GABA [6,7]. Current treatment options target this neurotransmitter imbalance.

### Symptoms and Consequences of Catatonia

Catatonia is a clinical syndrome characterized by a distinct constellation of psychomotor disturbances. A total of 2 subtypes have been described: retarded and excited [1,2]. The retarded subtype is characterized by symptoms such as immobility, staring, mutism, rigidity, withdrawal, and refusal to eat or drink, posturing, grimacing, negativism, waxy flexibility, echolalia or echopraxia, stereotypy, verbigeration, and automatic obedience. In contrast, excited catatonia is distinguished by pronounced psychomotor agitation, manifesting in behaviors such as aggression or impulsive behavior or movements, but may also encompass symptoms described earlier. If left untreated, both subtypes of catatonia may lead to increased morbidity and mortality. Complications include dehydration, muscle contractures, pressure ulcers, nutritional deficiencies, severe weight loss, thiamine deficiency, electrolyte disturbance, urinary tract infections, renal failure, rhabdomyolysis, pneumonia, and embolism [1]. Both subtypes may progress into malignant catatonia, a severe condition that may also occur acutely. Malignant catatonia is characterized by autonomic dysfunction and organ failure and may be fatal [8].

### Current Treatments

Rapid intervention in catatonia is crucial for achieving the most favorable prognosis [1]. At present, the primary pharmacological treatment involves administering a supratherapeutic dose of benzodiazepines, preferably lorazepam, over 3-7 days [1]. Lorazepam acts as a GABA-A receptor agonist, enhancing the inhibitory effects of GABA [9]. However, approximately 25% of patients do not completely respond to lorazepam treatment [1]. For these patients, electroconvulsive therapy (ECT) remains the only well-established alternative, demonstrating success rates of 80%-100% across various forms of catatonia, even when pharmacotherapy has failed [10]. Despite its efficacy, ECT is perceived as an “intrusive” treatment, compared to pharmacological options [11]. This perception often causes distress among caregivers of patients with catatonia, who may refuse this treatment option. Since communication with patients with catatonia is often challenging due to mutism, we seldom know the patients’ preferences about the treatment of catatonia. Concerns about ECT often revolve around potential cognitive side effects associated with ECT. The image of ECT in individual patient stories found on the internet or other media is usually not very positive, and the need for repeated general anesthesia during the typical 2- to 3-week treatment period, which usually involves 9-12 sessions, may cause concern among caregivers [11].

All parties, patients, caregivers, and health care professionals recognize the necessity of acute treatment for catatonia due to the possible severe consequences of the syndrome when left untreated. If patients and caregivers are unable to consent, and the patient faces serious medical risks, ECT may be administered under local compulsory mental health care acts or medical treatment agreements. However, an additional therapeutic option, such as sodium oxybate, could potentially reduce the need for compulsory treatment, or at least provide an additional option to make an informed decision.

### Rationale for Modification of GABA in Patients With Catatonia

Although the pathophysiologic mechanisms underlying catatonia are not fully elucidated, GABA likely plays an important role. Motor symptoms observed in catatonia are strongly linked to a deficient GABAergic state within the central nervous system (CNS) [2,6]. The GABA-model hypothesis of catatonia, supported by findings from several studies, suggests that the syndrome of catatonia is either due to reduced levels of GABA or dysfunction of GABA receptors in the CNS, resulting in decreased inhibitory signaling, which leads to signs of catatonia, such as stupor, catalepsy, mutism, mannerisms, stereotypy, agitation, echolalia, and echopraxia [12].

Functional imaging studies in humans have demonstrated that catatonia is associated with altered activity in the basal ganglia as well as in the orbitofrontal, prefrontal, parietal, and motor regions, suggesting that these cortical structures may also play a role in the pathophysiology of catatonia [13]. Most projection neurons within the basal ganglia are GABAergic, further supporting the role of GABA dysfunction in this condition [14]. This interpretation is further reinforced by observations that GABA-A binding is reduced in the cortical regions of patients with catatonia [6]. Motor and affective symptoms are correlated with these abnormalities in GABA-A binding. Furthermore, cortical abnormalities in patients with catatonia are normalized following exposure to lorazepam [3]. In addition, studies have revealed a decreased density of GABA-A receptors in patients with catatonia, further highlighting the significance of GABAergic mechanisms in the disorder [6].

### Current Treatment Options and GABA Modification

Lorazepam, the current primary treatment option for catatonia, binds to benzodiazepine receptors on the GABA-A ligand-gated chloride channel at several sites on neurons within the CNS. It enhances the inhibitory effects of GABA, resulting in an increased influx of chloride ions into the cell. The consequent hyperpolarization stabilizes the neuronal plasma membrane, enhancing cellular inhibition [9,15]. Lorazepam is therapeutically effective in approximately 75% of patients with catatonia [1].

The full working mechanism of ECT is not yet known. Nevertheless, ECT is widely recognized as highly effective in treating catatonia, even in the absence of randomized controlled evidence. A recent study by Breit et al [16] showed a mean reduction in BFCRS score of 82.8% after ECT treatment. An earlier study by Suzuki et al [17] found a recurrence rate of 63.6%, despite continuous pharmacotherapy.

A variety of studies have shown that ECT alters cerebral blood flow and glucose metabolism [18]. In addition, ECT has been found to affect the transmission of nearly all major neurotransmitters in the brain, including GABA [18]. During neurotransmission, ECT acts on various levels, including neurotransmitter synthesis, neurotransmitter release, binding of neurotransmitters to their receptors, as well as their reuptake [18,19]. Recent research shows a regional increase in brain volume post ECT, which is likely related to its neurogenic and synaptogenic effects [20]. Gbyl and Videbech [21] highlight that much remains unknown about the effect of ECT on neurochemical, neuroendocrine, synaptic, and cellular levels. However, it is strongly suggested that the likely deficit in GABA is restored after ECT [22].

### Findings From a Large Observational Study on Sodium Oxybate as a Treatment for Catatonia and Animal Studies

To our knowledge, there is only one large observational study that studies the effects of sodium oxybate as a treatment for various psychiatric and neurological disorders. This study included 288 participants diagnosed with conditions such as depression, anxiety, schizophrenia, epilepsy, and catatonia [23]. Sodium oxybate, the pharmacological agent used in this study, acts as a GABA-B receptor agonist and a precursor of GABA [24]. It was administered in doses ranging between 0.01 and 0.1 g/kg, 3 times daily either orally or intravenously (equivalent to 2.4-24 g/d for an 80 kg patient). Among patients with schizophrenia and catatonia, approximately 30 participants, the study showed a reduction of catatonic symptoms ranging from 60% to 70%. Specifically, there were improvements in anxiety, agitation, and restlessness.

Notably, these improvements were observed exclusively in patients with schizophrenia who exhibited catatonic symptoms or patients with disorganized schizophrenia (n=7), but not in patients with other psychiatric disorders (n=252), including those with depressive disorder, anxiety disorder, or schizophrenia of the paranoid type. Importantly, any patients participating in this study reported no adverse respiratory symptoms.

Findings from animal studies also suggest a strong involvement of a lack of GABA in catatonia. In a rat study, symptoms of catatonia such as muscular rigidity, catalepsy, and postural asymmetries could be produced by inhibition of striatonigral GABAergic neurons [25]. Similarly, another study found that the administration of GABA antagonists contributed to muscle rigidity [26]. Finally, animal studies showed that sodium oxybate contributed to an increase in GABA levels in the brain, possibly due to increased GABA-B receptor activity [27].

### Hypothesis and Aim

This study aims to provide robust evidence on the efficacy and safety of sodium oxybate for treating catatonia unresponsive to lorazepam, while also capturing the natural course and determinants of catatonia through its observational cohort. We hypothesize that pharmacological therapies, aimed at increasing GABA-B–mediated neurotransmission, could be effective in patients with lorazepam-resistant catatonia. We argue that sodium oxybate, the sodium salt of gamma-hydroxybutyric acid, is a viable alternative to lorazepam, which acts as a GABA-A agonist, because it is a precursor of GABA and acts as a GABA-B receptor agonist. Such a pharmacodynamic profile is expected to contribute to increased GABA-mediated neurotransmission [24,28] and could thus be beneficial to patients with catatonia.

If sodium oxybate proves to be an effective novel treatment for catatonia in patients with psychiatric conditions, this could have significant implications for the treatment of this condition. This new approach would expand the therapeutic options available to patients, their caregivers, and their treating physicians.

## Methods

### Study Design

The Laborit study is a multicenter trial combining an observational cohort study with an embedded randomized controlled trial (RCT). This design enables a comprehensive examination of catatonia symptoms, their progression, and potential determinants while also testing the efficacy of sodium oxybate as a treatment for catatonia in patients with psychiatric conditions unresponsive to a high-dose lorazepam regimen.

The name, Laborit, refers to Henri Laborit (1914-1995), a French surgeon who helped design the first antipsychotic drug, chlorpromazine. In addition, he extensively studied the clinical effects of sodium oxybate, after it was discovered in 1874 by chemist Alexander Zaytsev.

### Setting

The study will be conducted across multiple acute psychiatric wards and medical psychiatric units throughout the Netherlands. Study sites include the following hospitals: Leiden University Medical Centre, Amsterdam University Medical Centre, and Antes-Parnassiagroep, as well as mental health care institutions: GGZ inGeest, Arkin, and GGZ Centraal. These settings provide a broad catchment area necessary to recruit the required number of participants.

### Characteristics of Participants

The inclusion and exclusion criteria for the clinical trial part of the Laborit study are displayed in Textbox 1. For inclusion, catatonia has to be present, with a minimum score of 3 on the Bush Francis Catatonia Rating Scale as scored by the treating physician or trained nurse.

Exclusion criteria are based mainly on the need for rapid intervention in the treatment of catatonia (in case of malignant catatonia), or factors that cause high risk when taking sodium oxybate (sleep apnea, heart or renal impairment, and concurrent use of certain medications or alcohol). This study focuses on semiacute catatonic episodes caused by either psychotic or affective disorders; hence, chronic catatonia and patients with underlying somatic disorders are excluded. Due to the fact that antipsychotic-induced catatonia may often be treated by discontinuing the antipsychotic medication, patients who are likely to fall into this category are excluded from entering the trial part of the study [5]. They may be included in the observational part of the study.

**Inclusion and exclusion criteria.**

**Inclusion criteria**

- Age ≥18 years.
- Diagnosis of catatonia associated with unipolar depressive disorder, bipolar disorder, or psychotic disorder.
- Admitted to an acute mental health care ward or medical psychiatric unit.
- Inadequate response to escalating doses of lorazepam up to 24 mg/d over 4 days.

**Exclusion criteria**

- Chronic catatonia (duration >8 wks).
- Inability to take oral medication, unless hospitalized, where gastric tube administration is available.
- An underlying somatic condition causing catatonia is present or strongly suspected.
- Recent change in antipsychotic medication.
- History of antipsychotic-induced catatonia.
- Heart failure or renal impairment.
- Known sleep apnea.
- Use of specific medications (eg, GABAergic drugs, gamma-hydroxybutyric dehydrogenase inhibitors, and opioids other than tramadol).
- Current alcohol use disorder (alcohol use <7 d before starting the trial).
- Malignant catatonia present at inclusion or the development of malignant catatonia during the study, demanding immediate intervention.

### Power Calculation

To detect a clinically significant difference in remission rates (assuming 60% for sodium oxybate vs 15% for continued lorazepam) with a power of 0.80 and alpha of 0.05, 34 participants are needed. Allowing for a 20% dropout rate, 42 participants will be recruited. This sample size ensures sufficient power to test the primary outcome.

### Randomization

Patients will be randomly allocated to sodium oxybate or lorazepam. Patients will be randomized centrally, using random sequences of block sizes of 2, 4, and 6. We opt for this approach due to the low number of patients that will be obtained from each site, which hampers block randomization per site. The research support unit from the Department of Psychiatry, Amsterdam UMC, will be responsible for randomization using Castor Electronic Data Capture (EDC) software. Designated researchers will be responsible for randomization and communication of the allocated treatment with the treatment site. These researchers are not involved in data collection.

### Treatment Protocol

**Figure 1:**
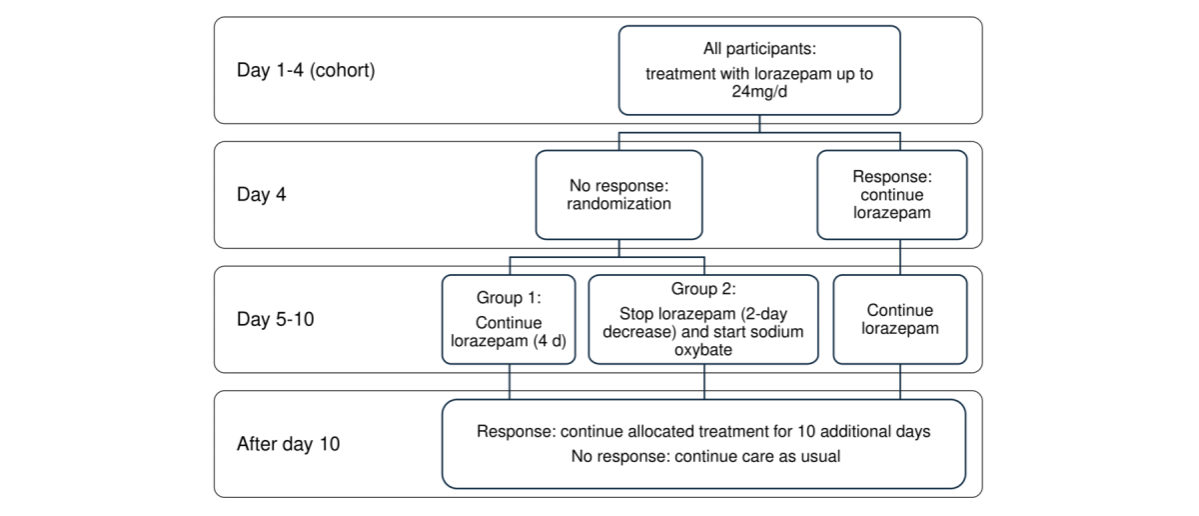
Overview of the treatment protocol.

When patients are enrolled in the study, the following steps, which are also displayed in Figure 1, will take place:

1. Patients enrolled in the cohort will receive lorazepam, titrated up to a maximum of 24 mg per day or up to the dose that causes sedation.
2. At the end of day 4, catatonia symptoms will be assessed using the Bush Francis Catatonia Rating Scale (BFCRS). If a reduction of less than 50% is achieved, patients may continue to enroll in the clinical trial part of the study.
3. When patients enroll, they will be randomized into two groups: treatment as usual, namely the continuation of high-dose lorazepam for 4 additional days, or a 60% reduction in lorazepam dosage over 2 days, followed by administration of sodium oxybate. Sodium oxybate will be titrated up to 27 g/day (4.5 g every 4 h).
4. If, after 4 days of treatment, patients improve more than 50% on the BFCRS, they may continue sodium oxybate treatment for an additional 10 days. If no response or remission has been achieved, patients will, in general, receive ECT. However, this decision lies outside the scope of this study and is a decision made between the treating professional, the patient, and his or her legal representative, in case of incapability.

At baseline in the cohort, we will collect descriptive data on the patient characteristics such as sociodemographic, clinical characteristics, somatic and psychiatric history, *DSM-5* (*Diagnostic and Statistical Manual of Mental Disorders* [Fifth Edition]) diagnosis, current and historical medication use, and the Clinical Global Impression (CGI) scale to assess illness severity, together with BFCRS, vital parameters and standard laboratory screening.

To ensure optimal homogeneity in the measurement of symptom severity, we have adjusted the original BFCRS to a new version. The original BFCRS contains 23 items on which between 0 and 3 points may be scored. However, the instructions for these different scores of severities are not well defined. In our adjusted BFCRS, we strive to decrease interrater variability by creating clear guidelines for scoring the severity of symptoms with every item of the list. We have created the adjusted BFCRS in collaboration with psychiatrists and nurses who are familiar with catatonia and use the original BFCRS in daily clinical practice. The adjusted list with the instructions may be found in Multimedia Appendix 1.

In the lorazepam group, patients will receive lorazepam 4 times daily, adhering to the standard treatment regimen for acute catatonia used in the participating wards. In the sodium oxybate group, medication will be administered every 4 hours, totaling 6 doses per day. Medication will be administered orally or through a gastric tube when available in case of admission to a medical psychiatric unit. BFCRS scores will be assessed 3 times a day by trained nurses or physicians on all wards. The research assistant will contact the wards where the participants are admitted 3 times a day. They will then receive information from the nurse or physician on call regarding the symptoms and BFCRS scores of the participant. The research assistant is sufficiently trained to determine if the BFCRS data are collected adequately by the ward staff.

The maximum dosage of sodium oxybate is 27 g per day, which amounts to 4.5 grams every 4 hours. For narcolepsy, for which sodium oxybate is a registered treatment option, the maximum dose is 9 grams per night in 2 equal doses 2.5 to 4 hours apart [29]. Opposed to patients with narcolepsy who only need this medication during the night, patients with catatonia need to be treated 24 hours a day. Because of this, we have extended the dosing regimen of narcolepsy, maintaining the same maximum dose every 4 hours. Table 1 shows the scheduled assessments per patient.

First-line laboratory screen: full blood count, C-reactive protein, renal, liver, and thyroid function tests, blood glucose, creatine phosphokinase, and iron measurement, and drug screen of urine. Repeated laboratory screening, as indicated by the treating physician. Tr: trial reduction of lorazepam. T0a: allocated treatment with lorazepam for 4 days. T0b: allocated treatment with sodium oxybate for 4 days after reduction of lorazepam. T1a: allocated treatment with lorazepam for 10 additional days, when lorazepam treatment contributed to an initial response. T1b: allocated treatment with sodium oxybate for 10 additional days.

**Table 1:** Timeline and schedule of assessments of patients in the Laborit study (based on SPIRIT [Standard Protocol Items: Recommendations for Interventional Trials] figure).

Assessment	Cohort study (Baseline, C1)	Cohort study (after 4 d of lorazepam, C4)	RCT^a^ (Tr)	RCT (T0a)	RCT (T0b)	RCT (T1a)	RCT (T1b)	Dropout or end of trial (T2)
Sociodemographics	✓							
Clinical characteristics	✓							
Somatic and psychiatric history	✓							
*DSM-5*^b^ diagnosis by a psychiatrist	✓							
*DSM-5* diagnosis through MINI^c^				✓	✓			
Medication use (current)	✓	✓						✓
Medication history	✓							
Clinical Global Impression scale	✓			✓	✓	✓	✓	✓
Bush Francis Catatonia Rating Scale	✓	✓	_✓_	✓	✓	✓	✓	
Physical + neurological examination	✓							
Blood pressure	✓	✓	_✓_	✓	✓	✓	✓	
Heart rate	✓	✓	_✓_	✓	✓	✓	✓	
Temperature	✓	✓	_✓_	✓	✓	✓	✓	
Oxygen level	✓	✓	_✓_	✓	✓	✓	✓	
Laboratory screening 1	✓	✓		✓	✓	✓	✓	
Assessment of side effects using SAFTEE^d^				✓	✓	✓	✓	

^a^RCT: randomized controlled trial.

^b^DSM-5: Diagnostic and Statistical Manual of Mental Disorders (Fifth Edition).

^c^MINI: Mini-International Neuropsychiatric Interview.

^d^SAFTEE: Systematic Assessment for Treatment Emergent Effects.

### Blinding

This study is single-blind, as patients may be aware of the allocated treatment group they are in. The research assistant responsible for data collection will be unaware of which treatment arm the participant is allocated to. Staff of the participating centers and participants are requested not to disclose whether patients were allocated to the sodium oxybate or lorazepam arm.

### Monitoring and Safety

Patients will be closely monitored for side effects, with particular attention to respiratory problems, through the continuous presence of an anesthesia technician for those receiving sodium oxybate in mental health care facilities. In hospitals, nurses will do this, and there will be backup from acute intervention teams from the Intensive Care Units. If patients exhibit side effects such as vomiting, they will be excluded from the trial, and treatment with sodium oxybate will be terminated due to the significant risk of aspiration associated with vomiting.

Participants are permitted to refuse the assigned medication once per day. However, if they refuse and subsequently miss more than 1 dose of the assigned drug, they will be excluded from the trial. The data that is gathered up until the moment of exclusion will be used in the intention-to-treat analysis. None of the medications will be administered forcibly under the previously mentioned laws. Given that this medication is an investigational product, individuals unable or unwilling to take the prescribed drug will be excluded from the trial and will receive necessary treatment with local guidelines and practices.

Following the guidelines of the CCMO (Dutch Central Committee on Research Involving Human Subjects), all suspected unexpected serious adverse reactions and serious adverse events (adverse events) will be documented and reported to the medical ethical review committee. In addition, a Data Safety and Monitoring Board (DSMB) has been installed to perform ongoing safety surveillance and to perform interim analyses on the safety data. This committee is an independent committee consisting of a psychiatrist with relevant experience in the treatment of catatonia, a statistician, and a pharmacologist familiar with the pharmacological characteristics of sodium oxybate.

### Primary and Secondary Outcomes

For both groups in the RCT, the BFCRS will be measured and documented 3 times daily. The primary outcome measure is the efficacy of sodium oxybate in reducing catatonia symptoms by 50%, as indicated by the BFCRS at the end of the fourth day of the allocated treatment, as compared to the last BFCRS measure before treatment. For the lorazepam group, this will be the BFCRS of the night shift of the last day of the cohort. For the sodium oxybate group, this will be the BFCRS of the night shift of the second day of lorazepam reduction. Secondary outcomes include remission rates (BFCRS <3 or no longer meeting *DSM-5* criteria for catatonia), the side effect profile of sodium oxybate compared to lorazepam, focusing on oxygen levels, blood pressure, heart rate, and vomiting, as well as the acute course of catatonia and its determinants in the observational cohort. Outcome evaluation methods are displayed in Table 2.

**Table 2:** Primary and secondary outcomes.

Outcome			Evaluation method
**Primary outcome**			
	Response to treatment	Difference in BFCRS^a^ between the start of treatment score and the last score after 4 days of treatment	
**Secondary outcomes**			
	Remission	No longer meeting *DSM-5*^b^ criteria of catatonia or BFCRS <3	
	Side effect profile	SAFTEE^c^	
	Acute course of catatonia	Determinants in the observational cohort	

^a^BFCRS: Bush Francis Catatonia Rating Scale.

^b^*DSM-5*: *Diagnostic and Statistical Manual of Mental Disorders* (Fifth Edition).

^c^SAFTEE: Systematic Assessment for Treatment Emergent Effects.

### Statistical Analysis

Statistical analyses will be performed on the intention-to-treat as well as the per-protocol sample. For all analyses, corrected *P* values of <.05 will be regarded as statistically significant. Safety data (eg, side effects) will be collected and summarized in the study results. A description of the analyses for the primary, secondary, and exploratory objectives is provided below.

#### Primary Outcome Measure Analysis

We will use a chi-square test to evaluate the response rate between the 2 groups after 4 days of treatment. Our study requires no adjustment of the type I error, given that it consists of 2 treatment groups and focuses on a single primary variable, response to treatment. The study follows a confirmatory statistical approach with a single, prespecified null hypothesis concerning the primary variable.

#### Secondary Outcome Measures Analysis

For the secondary outcome measures, we will first determine the remission after 4 days and subsequently after 10 days of treatment using logistic regression. Remission is defined as no longer meeting the *DSM-5* criteria of catatonia. We will also describe the percentage of side effects observed in participants assigned to high-dose lorazepam or sodium oxybate.

Comparative analyses between treatment groups will be conducted using *t* tests or their nonparametric equivalents for continuous variables and chi-square tests for categorical variables. Mixed-effects models will account for repeated measures and clustering within centers.

Second, in the cohort study, we will use standard clinical descriptive measurements from patient records to characterize the acute course of catatonia and its potential determinants, including sociodemographic and clinical characteristics. These characteristics encompass demographic information, additional *DSM-5* diagnoses beyond catatonia, severity and duration of catatonia, type of catatonia, the presence or absence of antipsychotic drugs before the onset of catatonia, and the effects of initial treatment with lorazepam. This analysis aims to identify distinct profiles of catatonia in acute psychiatric wards and their relationship to outcomes. This is particularly important given the current lack of studies that may predict which patients will improve from catatonia, with what treatment, and which are at risk of developing it. In addition, we will test the hypothesis that certain risk factors are associated with catatonia and describe their prevalence.

Third, we will test the hypothesis that patients with mild catatonia (eg, BFCRS <8) treated with both lorazepam and antipsychotics will have a higher remission rate compared to those treated with lorazepam alone. It is in line with clinical practice to continue antipsychotics in cases with mild catatonia.

We will use logistic regression to test this hypothesis, with antipsychotic use as a central determinant, and we will adjust for sociodemographic and clinical characteristics.

In addition, we will test the following hypotheses on possible risk factors associated with catatonia:

1. Patients who stop antipsychotics are at risk of developing catatonia. We will describe the prevalence of those with catatonia who stopped antipsychotic medication before the development of catatonia.
2. Patients who change antipsychotics are at risk of developing catatonia. We will describe the prevalence of those with catatonia who changed antipsychotic medication before the development of catatonia.
3. More patients are diagnosed with retarded catatonia than excited catatonia. In addition, we will describe the prevalence of patients with retarded catatonia (ie, characterized by immobility, mutism, staring, and rigidity) and patients with excited catatonia (ie, characterized by prolonged periods of psychomotor agitation).

Finally, we will analyze remission of catatonia in the patients enrolled in the cohort part of the study and treated with lorazepam alone. We will do so using logistic regression, adjusting for sociodemographic and clinical characteristics.

### Ethical Considerations

This study protocol was approved by the Institutional Ethics Board of Amsterdam UMC on March 22, 2023. Given the nature of catatonia, most patients will initially be unable to provide informed consent. Therefore, consent will be sought from legal representatives. All participants or their legal representatives, will provide verbal and written informed consent. Representatives will be informed about the trial alongside the information on the observational cohort in order to give them 4 days to consider the decision to participate. Patients regaining competence during the study will be asked to reconsent. Data from those withdrawing after reconsent will be included to minimize bias. Research data will be collected digitally using Castor EDC (a certified online deidentified data collection tool for medical research, password protected). For this study, a specific database has been created in Castor EDC and has been tested by several team members before data entry. At the point of inclusion into this study, a unique project number will be allocated to each subject, with no reference to initials or date of birth. The key to the data file is only available to the research assistants of the Amsterdam UMC and is password protected. Data entry is done by different research assistants for the cohort part and the trial part of the study. All subjects entering the trial part of the study receive a €25 (US $28.50) gift voucher.

## Results

This study was funded by the Dutch Brain Foundation (Hersenstichting) in December 2020. Correspondence of peer reviewers of this fund can be found in Multimedia Appendix 3. The study protocol was approved by the Institutional Ethics Board of Amsterdam UMC on 22-05-2023. As of March 2025, we have included the first 4 participants in the cohort part of the study, and we are awaiting the first trial enrollment. Please find the SPIRIT (Standard Protocol Items: Recommendations for Interventional Trials) checklist in Multimedia Appendix 2.

## Discussion

### Aim and Hypothesis

This study protocol outlines a multicenter RCT designed to evaluate the efficacy and safety of sodium oxybate for the treatment of catatonia that is unresponsive to lorazepam. In addition, it aims to capture the natural course and determinants of catatonia through an observational cohort. We hypothesize that sodium oxybate, by increasing GABA-B–mediated neurotransmission, could be effective in patients with lorazepam-resistant catatonia.

### Challenges

We anticipate possible difficulty with inclusion, as legal representatives of eligible patients may hesitate to enroll their relatives in our RCT due to concerns about the pharmacological product under investigation. Although sodium oxybate is an approved medication for treating narcolepsy, its association with gamma-hydroxybutyrate, a substance misused recreationally, may cause caution and concern.

Another challenge we face is the inability to conduct a double-blind study. Patients receiving sodium oxybate will be aware of their treatment due to the presence of an anesthesia technician assigned to monitor them during the initial 4 days, which will also be apparent to the treating staff on the wards. In addition, sodium oxybate may only be administered as a solution that needs to be taken orally, whereas lorazepam is available in tablets, intramuscular injection, and intravenous administration.

Although extensive exclusion criteria are necessary to ensure the safety of trial participants, they may introduce selection bias by excluding individuals with common comorbidities such as renal impairment. However, this is necessary to protect kidney function due to the high sodium levels in sodium oxybate. The decision to exclude participants with recent changes in antipsychotic drug use, which is known to induce catatonia, is based on the need for prompt intervention and discontinuation of these medications [5]. We expect catatonic symptoms to resolve in patients with antipsychotics–induced catatonia when medication is discontinued during the cohort phase of the study [5]. If participants have discontinued antipsychotic drugs during the observational phase of the study and do not recover from the catatonic symptoms, they are still eligible to enroll in the trial. In this way, we hope to minimize selection bias.

Although all teams of the different sites will be adequately trained and informed, the small number of inclusions across a relatively large number of study sites may cause fairly infrequent inclusions per site. However, we expect that the diversity and number of sites are necessary to be able to include enough participants to achieve the calculated statistical power for this study. Adherence to the study and treatment protocol must be well warranted.

Finally, we have based our dosing regimen of sodium oxybate on the current dosage used in the treatment of narcolepsy. However, we know that in the treatment of catatonia, lorazepam is used in supratherapeutic doses. Therefore, a possible challenge may be that our titration protocol for sodium oxybate will not use doses that are high enough to achieve a positive effect on catatonic symptoms, thus finding no effect of this treatment.

### Relevance

Catatonia is, in our view, an underexposed and underinvestigated syndrome with potentially serious complications. This may be due to numerous factors, such as lack of funding, inability to obtain informed consent from patients who are not competent during an episode of catatonia, or lack of interest from researchers and sponsors due to the low incidence of the syndrome in the general population. Our research could provide robust evidence of the efficacy of medication targeting GABA-B receptors in the treatment of catatonia.

### Conclusion

To conclude, our research, if positive, could provide patients with catatonia, their relatives, and health care professionals with a potential new treatment option in a regimen where current options are limited.

## Appendix

Multimedia Appendix 1 Bush Francis Catatonia Rating Scale adjusted for the Laborit study.

Multimedia Appendix 2 SPIRIT (Standard Protocol Items: Recommendations for Interventional Trials) checklist.

Multimedia Appendix 3 Peer-review report by the Dutch Brain Foundation (Hersenstichting).

## Abbreviations

BFCRS: Bush Francis Catatonia Rating Scale
CCMO: Centrale Commissie Mensgebonden Onderzoek
CGI: Clinical Global Impression
CNS: central nervous system
DSM-5: Diagnostic and Statistical Manual of Mental Disorders (Fifth Edition)
DSMB: Data Safety and Monitoring Board
EDC: Electronic Data Capture
ECT: electroconvulsive therapy
GABA: gamma-aminobutyric acid
RCT: randomized controlled trial
SPIRIT: Standard Protocol Items: Recommendations for Interventional Trials

## Data Availability

According to European law (General Data Protection Regulation), data containing potentially identifying or sensitive patient information is restricted. However, for academic researchers, data may be available upon request via the Labority study team at laborit@amsterdamumc.nl or through the corresponding author. We aim to publish the results of this study in a scientific journal.
