# Supplementary material for: Sodium Oxybate as a Potential New Treatment for Catatonia in Patients With Depression, Bipolar Disorder, or a Psychotic Disorder: Protocol for a Randomized Controlled Trial
Source: JMIR Res Protoc. 2025 Jul 24;14:e68356. doi: 10.2196/68356 (PMC12332447; doi:10.2196/68356)
Supplement: Multimedia Appendix 1 [file resprot_v14i1e68356_app1.pdf]

Algemene instructies bij afname BFCRS voor Laborit studie

- **Cohort**: BFCRS wordt één keer afgenomen op dag 1 en één keer op dag 4.
- **Trial**: BFCRS wordt driemaal per dag afgenomen: tijdens dag, avond en nachtdienst. Belmomenten staan in de tabel hieronder. De vitale parameters worden ook iedere dienst gemeten (dag, avond, nachtdienst)
- Zorg ervoor dat je **minstens 4 keer per dienst** contact hebt met de patiënt en observaties doet.
- Ga na invullen vragenlijst naar de **aftekenlijst** voorin de map en vul daar de datum, tijdstip, naam en paraaf in.

Scores dienen uitsluitend gebaseerd te zijn op gedrag geobserveerd gedurende het onderzoek, met uitzondering van de items 'teruggetrokkenheid' en 'autonome afwijkingen', die men zowel op direct geobserveerd gedrag als op (aantekening in) het patiëntdossier mag baseren. Om de onderdelen echopraxie/echolalie, stereotypie, maniërismen, verbigeratie, negativisme, impulsiviteit en automatische gehoorzaamheid te kunnen beantwoorden is het bij deze (aangepaste) versie van de BFCRS noodzakelijk om de patiënt gedurende de dienst 4 maal te observeren, en waar nodig vragen te stellen of te onderzoeken. De frequentie die gemeten wordt is afhankelijk van de hoeveelheid observaties waarbij het beschreven gedrag gezien wordt.

Als algemene regel geldt dat alleen items die duidelijk aanwezig zijn een score toegewezen dienen te krijgen; indien bij een item aanwezigheid niet duidelijk vast te stellen is, dient hier een score van '0' gegeven te worden. Indien een item niet af te nemen is, bijvoorbeeld door agressie of als iemand écht niet wakker gezien is, score 999 (geen waarde).

Onderzoeksprocedure:

1. **Activiteitsniveau, abnormale bewegingen, abnormale spraak** - Observeer de patiënt terwijl u pogingen onderneemt hem/haar in een gesprek te betrekken.
2. **Echopraxie** - De onderzoeker krabt zichzelf op overdreven wijze.
3. **Wasachtige buigzaamheid, gegenhalten, rigiditeit** - Onderzoek de arm op het tandradfenomeen. Poging tot herpositionering: instrueer de patiënt om de arm ontspannen te houden, beweeg de arm met afwisselend relatief weinig en veel kracht **-**
4. **Negativisme** - Geef de patiënt instructies om bepaalde handelingen uit te voeren, zoals: schud mijn hand, maak een vuist, wijs naar de deur, til je arm op.
5. **Mitgehen -** Vraag de patiënt de arm (voor zich uit) te strekken. Plaats één vinger onder zijn/haar hand en tracht deze langzaam verder omhoog te tillen na de instructie: 'Laat uw arm NIET door mij opheffen'.
6. **Ambitendentie -** Steek uw hand uit terwijl u het volgende zegt: 'Geef mij GEEN hand'.
7. **Automatisch gehoorzamen** - Steek uw hand in uw broekzak en zeg: 'Steek uw tong uit, ik wil er een naald in steken.' of 'Ik ga op uw teen staan.'.
8. **Grijpreflex** - Controleer of de grijpreflex aanwezig is.
9. Bekijk de aantekeningen van de afgelopen 24 uur in het patiënten rapport. Let daarbij in het bijzonder op orale inname, vitale tekens of enig incident.

Belplan voor trial:

Dienst	Tijden	Belmoment
Dag	08:00-15:00	14:00
Avond	15:00-23:00	22:00
Nacht	23:00-08:00	9:00 (overdragen aan dagdienst)

Nederlandse versie (BFCRS-N) - Aangepast voor de Laborit studie

M. Morrens, D. Schrijvers, H. Moens, B. Sabbe

	**Vraag**	**Antwoordopties**	**Antwoord/ Score**
1	**Opwinding**<br>Extreme hyperactiviteit, niet aflatende en schijnbaar doelloze motorische onrust. Niet toe te schrijven aan akathisie of doelgerichte agitatie.	0 = Afwezig<br>1 = Overmatig veel bewegingen<br>2 = Constant in beweging, hyperkinesie zonder rustperiodes<br>3 = Volledig ontwikkelde katatonische opwindingstoestand, eindeloze golf van tomeloze motorische activiteit **(gehele lichaam)**	
2	**Immobiliteit/stupor**<br>Extreme hypoactiviteit, onbeweeglijk, reageert nauwelijks op stimuli.	0 = Afwezig<br>1 = Zit ongewoon stil, gaat wel kortdurende interacties aan<br>2 = Praktisch geen interactie met de omgeving<br>3 = Stuporeuze toestand, reageert niet op pijnprikkels	
3	**Mutisme**<br>Reageert verbaal niet of alleen zeer beperkt.	0 = Afwezig<br>1 = Onduidelijk gefluister of beperkte verbale respons op de meeste vragen (>20 woorden/5 minuten)<br>2 = Spreekt minder dan 20 woorden/5 min<br>3 = Geen spraak	
4	**Staren**<br>Aanhoudende, starende blik, weinig of geen visuele verkenning van de omgeving, verminderd oogknipperen.	0 = Afwezig<br>1 = Weinig oogcontact, staart herhaaldelijk gedurende minder dan 20 sec voordat de aandacht wordt verplaatst, verminderd oogknipperen<br>2 = Starende blik die langer dan 20 sec wordt aangehouden, de aandacht wordt zelden verplaatst<br>3 = Aangehouden starende blik, reageert niet	
5	**Houding/katalepsie**<br>Houdt spontaan (een) houding(en) aan, inclusief alledaagse houdingen, blijft bijv. lange tijd achtereen zitten of staan zonder te reageren. Scoor de hoogste frequentie.	0 = Afwezig<br>1 = Minder dan 1 minuut<br>2 = Meer dan 1 minuut, minder dan 15 minuten<br>3 = Bizarre of banale houding die langer dan 15 minuten wordt aangehouden	

5*	**Is er sprake van**<br>A. Alleen houding (patiënt neemt actief een ongewone houding aan)<br>B. Alleen katalepsie (patiënt blijft in ongewone houding staan waarin hij passief is bewogen door onderzoeker)<br>C. Beide	○ A<br>○ B<br>○ C	
6	**Grimassen**<br>Het aanhouden van eigenaardige gelaatsuitdrukkingen.	0 = Afwezig<br>1 = Minder dan 10 seconden<br>2 = Minder dan 1 minuut<br>3 = Bizarre gelaatsuitdrukking(en) of een gelaatsuitdrukking die langer dan 1 minuut wordt aangehouden	
7	**Echopraxie/echolalie**<br>Het nabootsen van de bewegingen of spraak van de onderzoek(st)er. Scoor de hoogste frequentie, eventueel echopraxie en echolalie bij elkaar opgeteld. **(4 observaties per dienst)**	0 = Afwezig<br>1 = Sporadisch **(1 maal per dienst)**<br>2 = Frequent **(helft van observaties per dienst)**<br>3 = Voortdurend **(telkens)**	
7*	**Is er sprake van**<br>A. Alleen echopraxie (doet beweging onderzoeker na)<br>B. Alleen echolalie (spreekt onderzoeker na)<br>C. Beide	○ A<br>○ B<br>○ C	
8	**Stereotypie**<br>Repetitieve, <u>niet doelgerichte</u> motorische activiteit (o.a. vingerspel, zichzelf herhaaldelijk aanraken, klopjes geven of wrijven) waarbij de afwijking niet de beweging zelf is als wel de frequentie waarin deze wordt uitgevoerd. **(4 observaties per dienst)**	0 = Afwezig<br>1 = Sporadisch **(1 maal per dienst)**<br>2 = Frequent **(helft van observaties per dienst)**<br>3 = Voortdurend **(telkens)**	
9	**Maniërismen**<br>Eigenaardige, <u>doelgerichte</u> bewegingen (huppelen, op de tenen lopen, voorbijgangers groeten/salueren of overdreven karikaturen van banale bewegingen) waarbij de afwijking de activiteit zelf is. **(4 observaties per dienst)**	0 = Afwezig<br>1 = Sporadisch **(1 maal per dienst)**<br>2 = Frequent **(helft van observaties per dienst)**<br>3 = Voortdurend **(telkens)**	
10	**Verbigeratie**<br>Het herhalen van zinsneden of zinnen (zoals bij een haperende grammofoonplaat). **(4 observaties per dienst)**	0 = Afwezig<br>1 = Sporadisch **(1 maal per dienst)**<br>2 = Frequent **(helft van observaties per dienst)**<br>3 = Voortdurend **(telkens)**	
11	**Rigiditeit**<br>Het volharden in een verstijfde houding ondanks inspanningen om de patiënt te laten bewegen, uit te sluiten indien het tandradfenomeen (‘cogwheeling’) of tremor aanwezig is.	0 = Afwezig<br>1 = Geringe weerstand (**anders dan normaal)**<br>2 = Matige weerstand (**behoorlijke weerstand, kan nog wel gerepositioneerd)**	

		3 = Ernstige rigiditeit, kan niet gerepositioneerd worden<br>999 = niet kunnen onderzoeken	
12	**Negativisme**<br>Schijnbaar ongefundeerde weerstand tegen instructies of pogingen om de patiënt te bewegen/onderzoeken. Dit betekent tegendraads gedrag, doet precies het tegenovergestelde van wat gevraagd wordt.<br>Je kunt een aantal vragen stellen achter elkaar, bijvoorbeeld: schud mijn hand, til je arm op, wijs naar de deur, maak een vuist. Wanneer iemand compleet immobiel is kun je negativisme toetsen door te pogen de ogen van de patiënt te openen. **(4 observaties per dienst)**	0 = Afwezig<br>1 = Geringe weerstand en/of sporadisch tegenovergesteld gedrag **(1 maal per dienst)**<br>2 = Matige weerstand en/of frequent tegenovergesteld gedrag **(helft van observaties per dienst)**<br>3 = Hevige weerstand en/of onafgebroken tegenovergesteld gedrag **(telkens)**	
13	**Wasachtige buigzaamheid**<br>Bij het herpositioneren van de patiënt biedt de patiënt eerst weerstand alvorens toe te laten gerepositioneerd te worden, zoals bij het buigen van een kaars.	0 = Afwezig<br>3 = Aanwezig<br>999 = niet kunnen onderzoeken	
14	**Teruggetrokkenheid**<br>Weigert te eten, te drinken en/of oogcontact te maken.	0 = Afwezig<br>1 = Minimale orale inname/interactie gedurende minder dan 1 dag **(geen initiatief tot eten/drinken)**<br>2 = Minimale orale inname/interactie langer dan 1 dag **(moet actief aangespoord of geholpen worden)**<br>3 = Geen orale inname/interactie gedurende 1 dag of langer **(sondevoeding of infuus noodzakelijk)**	
15	**Impulsiviteit**<br>De patiënt vertoont plots onaangepast gedrag (begint bij voorbeeld door de gang te rennen, te roepen of zich uit te kleden) zonder aanleiding of uitlokking. Achteraf kan hiervoor geen of alleen een oppervlakkige verklaring gegeven worden. **(4 observaties per dienst)**	0 = Afwezig<br>1 = Sporadisch **(1 maal per dienst)**<br>2 = Frequent **(helft van observaties per dienst)**<br>3 = Voortdurend of niet bij te sturen **(telkens)**	
16	**Automatische gehoorzaamheid**<br>Het in overdreven mate voldoen aan het verzoek van de onderzoek(st)er of het spontaan doorgaan met uitvoeren van de gevraagde beweging. **(4 observaties per dienst)**<br>*Bij de 4 contactmomenten per dienst afwisselend vragen tong uit te steken en naald erin steken of vragen op teen te mogen staan.*	0 = Afwezig<br>1 = Sporadisch **(1 maal per dienst)**<br>2 = Frequent **(helft van observaties per dienst)**<br>3 = Voortdurend **(telkens)**<br>999 = niet kunnen onderzoeken	
17	**Mitgehen**.<br>‘Anglepoise lamp’ De arm wordt opgeheven als reactie op lichte druk door de vinger uitgeoefend, ondanks de instructie om dit niet te doen.	0 = Afwezig<br>3 = Aanwezig<br>999 = niet kunnen onderzoeken	

18	**Gegenhalten**<br>Weerstand tegen passieve beweging die in verhouding is met de sterkte van de stimulus die zich eerder automatisch dan uit vrije wil voordoet. Dus: hoe meer druk er gegeven wordt, hoe meer (tegen)druk er komt.	0 = Afwezig<br>3 = Aanwezig<br>999 = niet kunnen onderzoeken	
19	**Ambitendentie**<br>De patiënt lijkt motorisch ‘vast te zitten’ in (een) besluiteloze, aarzelende beweging(en).	0 = Afwezig<br>3 = Aanwezig	
20	**Grijpreflex**<br>Vast te stellen d.m.v. neurologisch onderzoek.	0 = Afwezig<br>3 = Aanwezig<br>999 = niet kunnen onderzoeken	
21	**Perseveratie**<br>De patiënt komt herhaaldelijk terug op hetzelfde gespreksonderwerp of volhardt in het uitvoeren van bewegingen.	0 = Afwezig<br>3 = Aanwezig	
22	**Vijandigheid**<br>Gewoonlijk op een indirecte manier en waarvoor achteraf geen of geen afdoende verklaring gegeven kan worden.	0 = Afwezig<br>1 = Haalt sporadisch uit **OF** met weinig kans op verwonding (1 x/24 uur)<br>2 = Haalt frequent uit **OF** met matige kans op verwonding (>1x/24uur)<br>3 = Vormt ernstig gevaar voor anderen **OF** er zijn maatregelen, naast medicamenteus ingrijpen nodig om dit te voorkomen ivm verwachte agressie denk aan fixatie of afzondering)	
23	**Autonome afwijkingen**<br>Kringloop: temperatuur (>37.5 of <36.5), bloeddruk (syst >140 of <100), hart/polsslag (>100 of <50) ademhalingsfrequentie (>20 of <10), overmatige transpiratie (beoordelen klinisch beeld)	0 = Afwezig<br>1 = Eén parameter wijkt af (uitgezonderd reeds bestaande hypertensie)<br>2 = Twee parameters wijken af<br>3 = Drie parameters wijken af	
		**TOTAAL**	

Ga na invullen vragenlijst naar aftekenlijst voorin de map en vul daar de datum, tijdstip, naam en paraaf in.
