# Supplementary material for: Sodium Oxybate as a Potential New Treatment for Catatonia in Patients With Depression, Bipolar Disorder, or a Psychotic Disorder: Protocol for a Randomized Controlled Trial
Source: JMIR Res Protoc. 2025 Jul 24;14:e68356. doi: 10.2196/68356 (PMC12332447; doi:10.2196/68356)
Supplement: Multimedia Appendix 3 [file resprot_v14i1e68356_app3.pdf]

Peer review report Hersenstichting (Dutch Brain Foundation):

Reviewer B-000067

Problem Definition, Objectives, and Intended Outcome

- **Is the problem clearly worded?**
  Good
- **Is the objective clearly worded?**
  Very good
- **To what extent do the project's objectives align with the objectives specified in the Brain Foundation Netherlands text?**
  Good
- **Does the project build on existing knowledge and practical experience?**
  Good
- **Is the intended result clearly worded?**
  Good
- **Is the project team fully aware of what follow-up steps are required?**
  Good
- **Is the intended result suitable to achieve the intended goal (are they doing the right things)?**
  Good

Extra remark: -

Plan of Action

- **Is the selected approach clearly worded?**
  Good
- **Is the target group involved, and have their points been incorporated into the project?**
  Good
- **Is the study design adequate? (e.g., does the chosen approach answer the set problem (are they doing things well))?**
  • Regarding study subjects: Is the study adequately powered? Is the inclusion feasible? Will the study subjects continue to the end?
  • For animal research: Is the chosen animal model suitable to advance the objective?
  • Are the primary and secondary outcome measures relevant?
  • To what extent are the proposed statistical methods sound?
  Good
- **Is the inclusion of patients/volunteers achievable within the intended time window?**
  Good

Extra remark:

The applicants mention that they want to exclude patients with malignant catatonia from the study, which makes sense. But how will the diagnosis of malignant catatonia be made? What are the criteria, and which parameters will be collected?
Patients treated with neuroleptic drugs will also be excluded. Many patients suffering from catatonia are on such drugs. What is the applicants' plan for those patients? Exclude them? Stop the neuroleptics?

Milestones, Budget, Project Team, and Risk Analysis

- **Is the milestone planning realistic?**
  Sufficient
- **Is the budget realistic?**
  Insufficient
- **How do the costs relate to the intended results?**
  Insufficient

- **Does the principal applicant have sufficient knowledge and experience for this project?**
  Good
- **How well-qualified is the project team in relation to the objective?**
  • Are the correct specialist groups involved?
  • Are the correct disciplines (academic, healthcare practice) involved?
  Very good
- **Are the risks clear?**
  Have the risks been realistically analyzed?
  Are the control measures adequate?
  Good

Extra remark: -

Budget:

- It's unclear why the PI (Principal Investigator) needs financial support.
- 0.3 FTE for the anesthesia technician seems very high, as the applicants state that about one patient will be treated per month.

Risk:
The chapter about risks was provided only in Dutch. Unfortunately, I couldn't fully understand it and therefore cannot comment on it.

Final Judgment
Honorable
This is an interesting application for a new treatment for patients suffering from catatonia. The research questions, the objective, and the methods are clear. The strengths of this project include the relevance of the topic to both medical and research fields, and the study team appears well-qualified to successfully conduct the study. However, there are some methodological shortcomings that I have addressed above.

Right of Reply
Response to the above review:
Dear Committee,
We thank the reviewer (B-000067) for the positive evaluation regarding the relevance and quality of the research proposal, as well as its feasibility. Based on the constructive feedback, we have made several clarifications and improvements to the previous proposal without significantly changing the design. In addition, we respond to other points of feedback. We follow the structure of the review in our response: 1) problem definition, 2) plan of action, 3) milestones, and 4) final judgment.

---

1. Problem Definition
The reviewer described this part of the study as good to very good and had no further comments.

---

2. Plan of Action
The reviewer asks for clarification regarding the diagnosis of malignant catatonia. This is a form of catatonia where the risk of mortality is even higher than in non-malignant catatonia [1-5]. Excluding this group of patients is essential. Due to the acutely life-threatening nature of malignant catatonia, these patients must be recognized in a timely manner so that treatment with ECT (electroconvulsive therapy) can be initiated as soon as possible (for these patients, ECT is administered after a maximum of two days of treatment with lorazepam) [1-5]. The new treatment we propose (sodium oxybate) is not suitable for patients with malignant catatonia.
We define malignant catatonia based on existing literature as follows [2-5]:

1. The presence of symptoms of non-malignant catatonia, and

2. The presence of fever or autonomic instability (fluctuating or elevated blood pressure, tachycardia, tachypnea, and excessive sweating), combined with moderately severe to severe rigidity.
3.

References:

1 Sienaert P, Dhossche DM, Vancampfort D, De Hert M, Gazdag G. A clinical review of the treatment of catatonia. *Front Psychiatry*. 2014;5:181.
2 Philbrick KL, Rummans TA. Malignant catatonia. *J Neuropsychiatry Clin Neurosci*. 1994;6:1-13.
3 *Malignant catatonia*. In: *Current Clinical Neurology: Movement Disorder Emergencies: Diagnosis and Treatment*, 2015 Springer chapter 5 malignant catatonia.
4 Sadr P, Hazeghazam M, Bailon M, et al. Management of a severe form of malignant catatonia: useful lessons in diagnosis and management, in Proceedings of the American Psychiatric Association Annual Meeting, San Francisco, Calif, USA, March 2013.
5 Strawn JR, Keck PE Jr, Caroff SN. Neuroleptic malignant syndrome. *Am J Psychiatry*. 2007;164:870-876.

The reviewer also asks for further clarification on the approach regarding patients using antipsychotics. The majority of studies indicate that antipsychotic use in patients with catatonia should be stopped to prevent the development of malignant catatonia [1]. There is often debate among psychiatrists about this, as a significant portion of patients have an underlying psychotic disorder, making continued treatment with antipsychotics seem logical. However, most studies show that patients with severe catatonia (Bush Francis Rating Scale ≥ 8) only recover adequately if antipsychotics are discontinued and treated simultaneously with lorazepam. For patients with mild catatonia (Bush Francis Rating Scale < 8), a combination of antipsychotics and lorazepam may be possible, provided the catatonia symptoms improve with this combination treatment [2]. As the patients participating in our trial have already received four days of lorazepam (with or without antipsychotics, for patients with mild catatonia) without improvement, it seems reasonable that, as is standard care, antipsychotics can be discontinued in these patients [1]. Our study follows the normal treatment of catatonia as much as possible [1].

References:

1 Sienaert P, Dhossche DM, Vancampfort D, De Hert M, Gazdag G. A clinical review of the treatment of catatonia. *Front Psychiatry*. 2014;5:181.
2 Strawn JR, Keck PE Jr, Caroff SN. Neuroleptic malignant syndrome. *Am J Psychiatry*. 2007;164:870-876.

3. Milestones

The reviewer is the only one who rated the budget as insufficient. This seems based on two points:

1. The reviewer does not understand why the principal investigator needs to be paid for this study.
2. The reviewer considers the number of FTE (Full-Time Equivalent) for the anesthesia nurse (0.38 FTE) to be high, given the expectation of including one patient per month.

We regret that we did not make it sufficiently clear why these costs are necessary.

Point 1)

It is unfortunately necessary for the principal investigator to request partial support from the Brain Foundation to carry out the study, as first-stream funding for the principal investigator is not sufficient to adequately fulfill all the tasks associated with this study. These tasks include coordinating the study across multiple institutions in the country, supervising the research nurse, ensuring the safety of the study, and training multiple teams to ensure consistency in recognizing and treating catatonia.

Point 2)
The reviewer indicates that the costs for the anesthesia nurse seem lower than 0.38 FTE. Unfortunately, this is not the case, and we will clarify why 0.38 FTE is necessary. With an inclusion rate of one patient per month, an anesthesia nurse must be present for at least four days (4x 24 hours) to ensure the safety of the patient. This corresponds to 12 shifts of 8 hours each, totaling 96 hours per month.
A total of 21 patients need to be monitored, which amounts to 96 x 21 = 2016 hours over the entire inclusion period. Currently, 2476 hours have been budgeted (181 weeks x 0.38 FTE x 36 hours). The 460 extra hours are needed to cover the costs for work

---

Reviewer B-000068
Problem statement, objective, and intended result

- Is the problem clearly worded?
  Good
- Is the objective clearly worded?
  Good
- To what extent do the project's objectives align with the objectives specified by the Brain Foundation Netherlands?
  Good
- Does the project build on existing knowledge and practical experience?
  Good
- Is the intended result clearly worded?
  Good
- Is the project team fully aware of the follow-up steps required?
  Good
- Is the intended result suitable to achieve the intended goal (are they doing the right things)?
  Very Good

Plan of approach

- Is the selected approach clearly worded?
  Good
- Is the target group involved and have their points been incorporated into the project?
  Very Good
- Is the study design adequate? (e.g., does the chosen approach address the problem well)? Regarding study subjects: Is the study adequately powered? Is the inclusion feasible? Will the study subjects continue to the end? For animal research: Is the chosen animal model suitable for advancing the objective? Are the primary and secondary outcome measures relevant? Are the proposed statistical methods sound?
  Good
- Is the inclusion of patients/volunteers achievable in the intended timeframe?
  Very Good

Milestones, budget, project team, and risk analysis

- Is the milestone planning realistic?
  Good
- Is the budget realistic?
  Good
- How do the costs relate to the intended results?
  Good
- Does the principal applicant have sufficient knowledge and experience for this project?
  Very Good

- How well-qualified is the project team in relation to the objective? Are the correct specialist groups and disciplines (academic, healthcare practice) involved?
  Very Good
- Are the risks clear?
  Very Good
- Have the risks been realistically analyzed?
  Very Good
- Are the control measures adequate?
  Very Good

Final judgment
Honorable

Response to the Reviewer
Dear Committee,
We thank the reviewer (B-000068) for their very positive evaluation of the entire research proposal. We will particularly respond to the comment made in the final judgment, as the reviewer made no further remarks.

Final judgment
The reviewer indicates that the application could have paid more attention to alternative pharmacological treatments for catatonia, such as baclofen, clozapine, or memantine.
We would like to clarify this point. Due to space constraints, we were previously unable to do so adequately.

1. We chose sodium oxybate because:
   - *Theoretical grounds*: There is substantial theoretical evidence supporting the expected positive effects of sodium oxybate in the treatment of catatonia, a point all reviewers enthusiastically support and consider highly relevant. For our rationale, please refer to pages 5-7 of our research proposal.
   - *Observational studies*: Strong evidence from observational studies suggests that sodium oxybate is effective in treating catatonia, while this is not known for baclofen. Additionally, there is extensive experience with sodium oxybate in terms of dosage, prior effects, safety, and its use in patient groups with narcolepsy. Baclofen, by contrast, has shown less therapeutic effect in narcolepsy than sodium oxybate, making it more logical to test sodium oxybate rather than baclofen [1,2]. Furthermore, there are no data on the effect of baclofen in patients with catatonia.
2. *Clozapine*: It is indeed a good alternative for treating catatonia in patients with an underlying psychotic disorder. The reviewer, Professor Tarun Bastiampillai, has published two important studies on this subject [3,4]. Theoretically, clozapine could, like sodium oxybate, stimulate GABA neurotransmission and thus reduce catatonia. However, the direct effect of clozapine as a GABA-B agonist has been less studied compared to sodium oxybate. The two main drawbacks of treating catatonia with clozapine are:
   - *Duration of treatment*: We expect that sodium oxybate will lead to a significant reduction in catatonia within a few days, while reaching a maximum dose with clozapine takes 5 weeks [5], which increases the risk of morbidity and mortality associated with prolonged exposure to catatonia [6,7]. This would also necessitate longer compulsory treatment, which is not desirable for the patient or their family. An RCT with clozapine, while a shorter treatment duration is possible, seems disproportionate for this reason.

- *Risk of malignant catatonia*: Treatment with clozapine in catatonia patients appears to be associated with a greater risk of developing malignant catatonia [8-11], an extremely undesirable and harmful side effect.

3. *Memantine* and other glutamate antagonists, such as amantadine, are also potential treatments for catatonia. The hypothesis is that catatonia involves a GABA-glutamate imbalance [6]. In our grant application, we focus on restoring GABA metabolism, given the strong evidence for the efficacy of lorazepam, sodium oxybate, and ECT in affecting GABA metabolism. There is limited evidence that inhibiting glutamate metabolism might also be effective. Findings suggesting the efficacy of memantine or amantadine are based on case reports, many of which involved patients treated with high doses of antipsychotics [12-16]. In these studies, the antipsychotic was stopped when glutamate antagonists were started, leaving it unclear which intervention reduced the catatonic symptoms.

In an ideal world, we would conduct a more elegant RCT, randomizing patients between lorazepam, memantine, or sodium oxybate. This would test the effectiveness of these treatments while providing more clarity on the pathophysiological mechanism of catatonia. However, the reality is that several clinical centers across North Holland are already required, and adding another arm to the RCT would significantly increase the costs, which is not feasible within the current budget.

Literature:
1 Carter LP, Koek W, France CP. Behavioral analyses of GHB: receptor mechanisms. Pharmacol Ther. 2009;121:100-114.
2 Vienne J, Bettler B, Franken P, Tafti M. Differential effects of GABAB receptor subtypes, {gamma}-hydroxybutyric Acid, and Baclofen on EEG activity and sleep regulation. J Neurosci. 2010;30:14194-14204.
3 Nair PC, McKinnon RA, Miners JO, Bastiampillai T. Binding of clozapine to the GABAB receptor: clinical and structural insights [published online ahead of print, 2020 Mar 13]. Mol Psychiatry. 2020;10..
4 Lander M, Bastiampillai T, Sareen J. Review of withdrawal catatonia: what does this reveal about clozapine?. Transl Psychiatry. 2018;81:139.
5 https://www.clozapinepluswerkgroep.nl/publicaties/richtlijn-voor-het-gebruik-van-clozapine/
6 Sienaert P, Dhossche DM, Vancampfort D, De Hert M, Gazdag G. A clinical review of the treatment of catatonia. Front Psychiatry. 2014;5:181.
7 Pelzer AC, van der Heijden FM, den Boer E. Systematic review of catatonia treatment. Neuropsychiatr Dis Treat. 2018;14:317-326.
8 Lee JW. Catatonic variants, hyperthermic extrapyramidal reactions, and subtypes of neuroleptic malignant syndrome. Ann Clin Psychiatry. 2007;19:9-16.
9 Paparrigopoulos T, Tzavellas E, Ferentinos P, Mourikis I, Liappas J. Catatonia as a risk factor for the development of neuroleptic malignant syndrome: report of a case following treatment with clozapine. World J Biol Psychiatry. 2009;10:70-73.
10 DasGupta K, Young A. Clozapine-induced neuroleptic malignant syndrome. J Clin Psychiatry. 1991;52:105-107.
11 Strawn JR, Keck PE Jr, Caroff SN. Neuroleptic malignant syndrome. Am J Psychiatry. 2007;164:870-876.
12 Carroll BT, Goforth HW, Thomas C, Ahuja N, McDaniel WW, Kraus MF, et al. Review of adjunctive glutamate antagonist therapy in the treatment of catatonic syndromes. J Neuropsychiatry Clin Neurosci 2007;19:406–12.10.
13 Obregon DF, Velasco RM, Wuerz TP, Catalano MC, Catalano G, Kahn D. Memantine and catatonia: a case report and literature review. J Psychiatr Pract 2011;174:292–9.
14 Babington PW, Spiegel DR. Treatment of catatonia with olanzapine and amantadine. Psychosomatics (007;48:534–6

15 Hervey WM, Stewart JT, Catalano G. Treatment of catatonia with amantadine. Clin Neuropharmacol 2012;352:86–710.
16 de Lucena DF, Pinto JP, Hallak JE, Crippa JA, Gama CS. Short-term treatment of catatonia with amantadine in schizophrenia and schizoaffective disorder. J Clin Psychopharmacol 2012;324:569–7210.

B-000069
Problem definition, objective, and intended result

- **Is the problem clearly worded?** Very good
- **Is the objective clearly worded?** Very good
- **To what extent do the project's objectives align with the objectives specified in the Brain Foundation Netherlands text?** Good
- **Does the project build on existing knowledge and practical experience?** Very good
- **Is the intended result clearly worded?** Very good
- **Is the project team fully aware of what follow-up steps are required?** Very good
- **Is the intended result suitable to achieve the intended goal (are they doing the right things)?** Very good

This is a timely and excellent topic. Lorazepam treats the majority of patients with catatonia, but some who do not respond may be treated with ECT, which is highly effective but can cause transient cognitive side effects. Establishing an alternative medication such as oxybate is very reasonable.

Plan of approach

- **Is the selected approach clearly worded?** Very good
- **Is the target group involved and have their points been incorporated into the project?** Very good
- **Is the study design adequate?** (e.g., does the chosen approach address the problem effectively? Is it well powered? Feasibility of inclusion? Continuation of subjects?) Very good
- **Is the inclusion of patients/volunteers achievable in the intended time window?** Very good

Some questions about the design and method:

1. The patients are not blind to the treatment, but will the raters assessing catatonia be blinded to the treatment condition?
2. Catatonic patients are often withdrawn and negativistic, with reduced oral intake. Are the researchers confident that patients can take oral medications several times a day? What is the alternative plan if oral dosing is not feasible—remove the patient from the study, or use IV/nasogastric methods?
3. The main outcome is a 50% drop on the catatonia scale. Is this measured from the initial baseline or after the lorazepam trial? If the patient has a partial response (e.g., 25% drop), will the oxybate effect be measured from this new score or from the baseline four days prior? It seems the effect should only apply after the oxybate is administered, so the "new" score should be used.

Milestones, budget, project team, and risk analysis

- **Is the milestone planning realistic?** Very good
- **Is the budget realistic?** Very good
- **How do the costs relate to the intended results?** Very good
- **Does the principal applicant have sufficient knowledge and experience for this project?** Good
- **How well-qualified is the project team in relation to the objective?** Very good
- **Are the risks clear?** Very good
- **Have the risks been realistically analyzed?** Very good
- **Are the control measures adequate?** Very good

Final judgment

- **Honorable**

Dear committee,
We thank the reviewer (B-000069) for their very positive evaluation of the entire research proposal. We will focus our response on the comments regarding the plan of approach since the reviewer raised three questions in that section. Otherwise, the reviewer has no comments beyond their positive scores.

Plan of approach

1. Both this reviewer and reviewer B-000070 emphasize the importance of having blinded researchers carry out the assessments without knowing the randomized treatment condition. We agree with this and will certainly ensure it is done once the study begins. We regret not having described this more thoroughly in the original plan.
2. The reviewer notes that some catatonic patients may struggle with fluid, food, and medication intake, which could make oral administration of sodium oxybate unfeasible. We agree that an alternative plan for medication administration, aside from excluding these participants, must be explored. One option is inserting a feeding tube for patients who agree to it. We anticipate that this will be an option for some catatonic patients and will consult our group of experts. Unfortunately, administering intravenous sodium oxybate is not feasible, as no IV preparation is available.
3. The reviewer notes uncertainty about when the response (a 50% reduction in catatonia symptoms) should be measured. The reviewer prefers that the response is measured after randomization, i.e., when the intervention starts, with the use of sodium oxybate compared against lorazepam. We agree with the reviewer and thank them for the opportunity to clarify this point.

B-000070
Problem definition, objective, and intended result

- **Is the problem clearly worded?** Very good
- **Is the objective clearly worded?** Very good
- **To what extent do the project's objectives align with the objectives specified in the Brain Foundation Netherlands text?** Very good
- **Does the project build on existing knowledge and practical experience?** Good
- **Is the intended result clearly worded?** Very good
- **Is the project team fully aware of what follow-up steps are required?** Good
- **Is the intended result suitable to achieve the intended goal (are they doing the right things)?** Adequate

This is a very interesting and important approach, addressing unmet needs in a critical condition. Catatonia remains a challenge due to its high mortality. Currently, lorazepam and ECT are the only treatment options, but some patients or caregivers reject ECT. Therefore, sodium oxybate could be a potential treatment for acute catatonia. Patient participation in the study planning is excellent.
Plan of approach

- **Is the selected approach clearly worded?** Very good
- **Is the target group involved and have their points been incorporated into the project?** Very good
- **Is the study design adequate?** Adequate
- **Is the inclusion of patients/volunteers achievable in the intended time window?** Very good

The feasibility of the study is high, with a randomized allocation of subjects. The outcome measures are great, but secondary outcomes could be extended to include functioning, quality of life, illness severity, and concomitant medication use. To improve rigor, consider including measures for screening catatonia by the investigators, with blinded raters. Blinding on the patients' side is less critical but would be more challenging.
Uncertainties include:

- Why are antipsychotic drugs excluded? This may reduce eligible subjects with psychosis.
- How are recurrent catatonia episodes handled?
- How is malignant catatonia defined?
- Why not use DSM-5 catatonia criteria?
- How will dropouts be managed in the statistics? Why not use the last-observation-carried-forward method for the ITT population?

Milestones, budget, project team, and risk analysis

- **Is the milestone planning realistic?** Very good
- **Is the budget realistic?** Very good
- **How do the costs relate to the intended results?** Very good
- **Does the principal applicant have sufficient knowledge and experience for this project?** Good
- **How well-qualified is the project team in relation to the objective?** Good
- **Are the risks clear?** Very good
- **Have the risks been realistically analyzed?** Very good
- **Are the control measures adequate?** Adequate

The PI and most of the team have a neuroscience background and experience with the substance but limited experience with the specific condition. The two psychiatrists have clinical experience with catatonia. Including safety measures such as regular side effect screenings is important.

Final judgment

- **Honorable**

This study plan addresses an urgent clinical problem with extraordinary relevance to the field. Catatonia is life-threatening, with limited treatment options. The idea is novel, based on promising open-label study results. If successful, this study could be a game changer, even internationally. However, some adjustments to the study design are necessary to ensure scientific rigor. Without these, even very good results may not be fully appreciated, especially if it remains an unblinded study.

Dear Committee,

We thank the reviewer (B-000070) for the very positive assessment of the entire research proposal. Based on the constructive feedback, we have made several clarifications and improvements to the previous proposal, without significantly altering the design. In addition, we are responding to the other points raised in the feedback. Our response follows the structure of the review: 1) problem statement, 2) plan of approach, 3) milestones, and 4) final evaluation.

1. Problem Statement

No points for improvement were identified in the problem statement section.

2. Plan of Approach

The reviewer also rates the plan of approach as very good and discusses some possible areas for improvement.

The reviewer indicates that the study, in its current setup, is highly feasible and is satisfied with the primary outcome measure. The reviewer suggests expanding the number of secondary outcome measures to include assessments of functioning and quality of life of participants, as well as descriptions of medication use and the severity of different patients with catatonia.

We agree that these are important additional secondary outcome measures, and we propose to add the following aspects:

- We will use the **World Health Organization Disability Assessment Schedule 2.0 (WHODAS 2.0)** [1] to assess functioning at the baseline measurement (start of the intervention) and after the intervention. The patients' nurses will be interviewed by a blinded researcher, who will not be aware of the randomized treatment condition, ensuring no additional burden on the patients.

- This blinded researcher will also record medication use before the catatonic episode and during the intervention. Additionally, the **Clinical Global Impression Scale** [2] will be used to estimate changes in the severity of the clinical picture.
- Lastly, we will assess the intervention's effect on quality of life using the **EQ 5D 5L** proxy version for caregivers [3], as direct assessment of quality of life by the patient is challenging during a catatonic episode, making it unlikely that sufficient data could be collected without these proxy versions. Using these proxy versions also ensures no additional burden on the patient.
-

Furthermore, the reviewer stresses the importance of having a blinded researcher assess the level of catatonia, which we have already agreed to with reviewer B-000069 in the plan of approach section. We take this opportunity to clarify this point further.
The reviewer, like reviewer B-000067, asks why patients using antipsychotics are excluded. We refer to our response to reviewer B-000067, plan of approach section, for the answer to this question. The reviewer notes that excluding these patients might reduce the number of participants in the study. We have accounted for this in our study design by including a large catchment area. Most acute inpatient clinics from the three largest healthcare providers in North Holland are participating (GGZ Noord-Holland-Noord, Arkin, and GGZ inGeest).
Another question from this reviewer is how to handle patients with recurring catatonic episodes. Patients can only be included in the study once. We will register whether participants have a history of recurring catatonic episodes. The reviewer also asks, like reviewer B-000067, how malignant catatonia is defined. For the answer to this question, we refer to our response to reviewer B-000067 in the plan of approach section.

Additionally, the reviewer suggests not diagnosing patients with catatonia using the **Bush Francis Catatonia Rating Scale** (cut-off point greater than two). We appreciate the reviewer's input on improving the research proposal and will adopt the suggestion to use the **DSM-5** diagnostic criteria for catatonia. Initially, we chose to use only the Bush Francis Catatonia Rating Scale because it is the most commonly used clinical tool for describing catatonia, and clinicians are primarily interested in how catatonia symptoms change over time. However, we agree with the reviewer that it is better to establish the diagnosis using the DSM-5 criteria and then measure the severity of catatonia using the Bush Francis Catatonia Rating Scale. This also provides the opportunity to investigate whether the Bush Francis Catatonia Rating Scale is suitable for diagnosing catatonia according to the DSM-5, something that is currently assumed but not tested.

Lastly, the reviewer asks how we will handle dropouts and suggests using an **Intention-to-treat (ITT)** method such as **Last-Observation-Carried-Forward (LOCF)** for handling missing data due to dropouts. When calculating the sample size, we accounted for a loss of power due to dropouts. We had planned to include extra patients to compensate for a 20% dropout rate. However, in our analysis plan, we had not yet addressed potential bias from selective dropout. We thank the reviewer for this suggestion. To prevent bias due to selective dropout, we will use an **ITT analysis strategy** [4], which includes all randomized individuals. In this strategy, we will use a main analysis that is valid under a plausible assumption for the missing data. We prefer a **Missing-At-Random (MAR)** assumption over the assumption underlying LOCF. After the main analysis, we will conduct sensitivity analyses to explore how the effect estimates would change if the MAR assumption is violated. LOCF will be included as one of the sensitivity analyses.

References:

1. WHODAS 2.0 - Proxy Version
2. Busner J, Targum SD. The clinical global impressions scale: applying a research tool in clinical practice. Psychiatry. 2007;4:28-37.
3. EQ-5D-5L Proxy Versions

4. White IR, Carpenter J, Horton NJ: Including all individuals is not enough: lessons for intention-to-treat analysis. Clin Trials. 2012, 9: 396-407.

3. Milestones
The reviewer emphasizes the importance of safety measures and side effect monitoring. The reviewer from the patient panel (B-000094) indicates that the project handles risks, safety, and side effect monitoring very well, with regular screenings for side effects. We regret that we completed the risk analysis in Dutch, which may have led some reviewers to not fully assess this part. However, in the English text, we did mention that during treatment with sodium oxybate, an anesthesiology nurse will be present 24 hours a day for four days to address any life-threatening side effects. Additionally, side effects will be monitored daily during the study, starting with measuring autonomic dysregulation (blood pressure, heart rate, temperature, and oxygen saturation) three times a day. We will also systematically monitor side effects using the **Systematic Assessment for Treatment Emergent Events-Specific Inquiry (SAFTEE-SI)** [1].

References:
1. Levine J, Schooler NR. SAFTEE: a technique for the systematic assessment of side effects in clinical trials. Psychopharmacol Bull 22: 343-381.

4. Final Evaluation
The reviewer notes that this is an important and highly relevant study, with "extraordinary relevance in the field." The points for improvement summarized in the final evaluation have been addressed above. We also reiterate the importance of having a blinded researcher perform the assessments, as indicated by the reviewer.

B-000094
Is the project relevant from the perspective of the target patient group? Consider:
- Fulfillment of the needs of patients/target group
- Improvement in health
- Improvement in quality of life
- Improvement in social participation and self-reliance
- Improvement in general care

  Good

Extra remark: This research seems very relevant for this target group because catatonia is a very serious condition with a significant disease burden, and currently there are only two treatment options. The transition to ECT is a major step with many unpleasant side effects for the patient. Moreover, a recent meta-analysis shows that ECT is not effective. If this research shows that Sodium Oxybate can prevent the need for ECT, it would positively impact general care.
It is not entirely clear whether catatonia is a one-time episode or not. Is there also attention to the causes of catatonia? This might provide insight into how to better manage catatonia.
Because it is rare, it is difficult to study, but it also raises questions because it involves such a specific group. Perhaps discussions with former clients and their families could provide valuable additional information and recommendations.

Is the plan of approach feasible from the patient/participant's perspective? Consider:
- The burden on participants during activities (surveys, tests, treatment, etc.)
- The burden on participants over the course of the entire study (number of moments)
- Possible objections from patients/participants against participation (e.g., preference for a particular treatment)

  Good

Extra remark: There are risks for the patient, but due to the severity of the condition, there are really no other options. The risks have been carefully considered and addressed, such as administering extra oxygen. On the other hand, current care (ECT) is even more burdensome.
It is important that the patient's family is well-informed about the risks associated with the treatment.

Are the involved participants sufficiently facilitated? Consider:

- Time, travel reimbursement, skill development
  Insufficient

Extra remark: There is no mention of compensation, such as travel costs for participants, or anything being done for family members. While experts by experience were involved in the design, their involvement in the execution is not evident in the proposal. Perhaps in this research, the use of experts by experience is neither possible nor necessary, as the outcome concerns life or death rather than quality of life. But it would have been good to mention this in the research proposal. Since it is an involuntary setting, extra attention to the family or loved ones would be beneficial.
When organizing a focus group, it is important to use an external facilitator, which is not mentioned. It might also be good to actively seek input from those dissatisfied with current treatment, possibly through an advertisement.

Is there attention to communicating the project and results? Consider:

- To project participants
- To the (future) target group
  Good

Extra remark: The participants themselves may not be able to communicate, but ensure good communication with their families. For example, develop a brochure for them.
Additional remarks:

What recommendations would you give the project team to optimize the project from the patient/target group's perspective?

- Hold discussions with former clients/family/loved ones to gather more information and feedback from that perspective.
- When organizing a focus group, have the conversation led by an independent facilitator.
- Actively seek out individuals dissatisfied with current treatment, possibly via an advertisement.
- Include an amount in the project proposal for reimbursement of participants' or family members' expenses.
- Pay specific attention to family and loved ones in the research.
- Involve more experts by experience/family members in the research, or explain in the proposal why their involvement is not necessary (if there is a reason for this).
- Ensure good information is provided to family members/loved ones. For example, develop a brochure or other written material.
- Organize patient/family journey sessions, if possible, to gain a broader understanding of the situation and all it entails. Also, assess the burden.

It may also be worth investigating the exact cause of catatonia in future research. Are there factors that trigger catatonia? By understanding these causes, interventions and treatments can be better targeted (prevention).

Response
Dear committee,
We thank the reviewer (B-000094) for the very positive evaluation of the entire research proposal. Based on the constructive comments, we have made a number of clarifications/improvements to the

previous proposal without significantly changing the design. We will structure our response as follows: 1) relevance from the perspective of the patient group/target group, 2) feasibility from the patient/participant's perspective, 3) facilitation of the involved participants, 4) attention to communication of the project and results, and 5) recommendations from the patient panel to the project team.

1) Relevance from the patient group's perspective

The reviewer points out that this is a highly relevant study.
The patient panel is unclear whether catatonia is a one-time episode. We did not describe in the Dutch text that catatonia is not episodic but can persist for a long time if no acute treatment is provided. We will clearly describe in the information material for patients and families what a catatonic state looks like and the consequences of treating or not treating it.
The reviewer asks whether there is attention to the causes of catatonia. This is an important question. The hypothesis is that there is a GABA-glutamate imbalance in catatonia [1-3]. It is believed that in cases of severe depression or psychosis, this imbalance occurs, and catatonia is present in 10% of psychiatric inpatients. To prevent catatonia, it is important to adequately treat the patient's psychiatric condition (depression/bipolar disorder or psychosis).
To treat catatonia, it is crucial to restore the GABA-glutamate balance. As mentioned in the application, this can be done by restoring GABA metabolism through lorazepam or ECT. In our application, we also want to investigate whether this can be achieved with sodium oxybate. If so, this would be groundbreaking not only for understanding catatonia but also for expanding treatment options.
The reviewer also notes that discussions with former clients and families may provide valuable additional information and recommendations. Besides the former clients and their families from GGZinGeest who gave recommendations for the study's design, we will invite other former clients and their families to monitor the study and provide input on the interpretation and dissemination of the findings. The reviewer further notes that catatonia is uncommon. However, international research and clinical practice indicate that this is not true. As mentioned, catatonia is present in 10% of psychiatric inpatients but often goes unrecognized due to inadequate training among nurses and psychiatrists. It also frequently occurs in somatic hospitals, where it is even less recognized. Raising awareness of the identification and treatment of catatonia is crucial, and this study aims to contribute to that effort.

2) Feasibility from the patient/participant's perspective

The reviewer highlights the importance of informing the patient's family about the risks involved in the treatment. This is, of course, a critical point. We recognize that this is essential for the success of the study, and we will do so with input from families and former clients. The patient panel's recommendations, such as increasing the group of former clients and their families and having an external facilitator lead discussions, will be adopted. Although conversations with former clients and their families were indeed held about the study's design, we regret not mentioning this in the Dutch version of the research application.

3) Facilitation of the involved participants

Finally, it is noted that extra attention should be given to family or loved ones in this study. We take this to heart and will regularly speak with families about the course of the treatment and reimburse travel expenses for this. We will inform family members at least once a week, either face-to-face or via video calls, about the progress of the patient's catatonia. If there is a need for more support, we will of course facilitate it. In clinical practice, we always involve family members intensively. Furthermore, travel costs will be reimbursed as recommended.

4) Attention to the communication of the project and results

The reviewer suggests providing proper information to patients and their families, including through

a brochure. We take this point to heart and will provide extensive information about the study, including the possible benefits and risks. We will support this, as suggested, with materials such as brochures for the patient and their family.

5) Recommendations from the patient panel to the project team

The following recommendations are made by the reviewer from the patient panel, summarizing the suggestions/improvements previously mentioned:

- Hold discussions with former clients/families/loved ones to gather more information and feedback from that perspective.
- When organizing a focus group, have the conversation led by an independent facilitator.
- Actively seek input from dissatisfied individuals, possibly via an advertisement.
- Include an amount in the project proposal for reimbursing participants' or family members' expenses.
- Pay specific attention to family and loved ones in the research.
- Involve more experts by experience/family members in the research or explain why they are not involved (if applicable).
- Provide clear information to family members/loved ones. For example, develop a brochure or other written material.
- Organize patient/family journey sessions to gain a broader understanding of the situation and assess the burden.

We are grateful to the reviewer for these practical suggestions, and we will implement all of them in our research.

Finally, the reviewer mentions the importance of investigating the causes of catatonia, and we refer to our response under "1) Relevance from the patient group's perspective."

References:

1 Sienaert P, Dhossche DM, Vancampfort D, De Hert M, Gazdag G. A clinical review of the treatment of catatonia. Front Psychiatry. 2014;5:181.
2 Dhossche DM, Sienaert P, van der Heijden FM. Mechanismen van katatonie. Tijdschr Psychiatr. 2015;57:99-103.
3 Fink M, Taylor M. Catatonia. A clinician’s guide to diagnosis and treatment. Cambridge: University Press 2003
